# Oral health status, on-ice and off-ice test performance, and match performance in elite male ice hockey players: a pilot study

**DOI:** 10.3389/froh.2025.1725259

**Published:** 2026-01-05

**Authors:** René Schwesig, Eduard Kurz, Richard Brill, Stephan Schulze, Andreas Wienke, Christian Ralf Gernhardt

**Affiliations:** 1 Department of Orthopaedic and Trauma Surgery, Martin-Luther-University Halle-Wittenberg, University Medicine Halle, Halle, Germany; 2 Department for Diagnostic and Interventional Radiology, University Hospital Halle (Saale), Halle, Germany; 3 Institute of Medical Epidemiology, Biostatistics and Informatics, Martin-Luther-University Halle-Wittenberg, University Medicine Halle, Halle, Germany; 4 University Outpatient Clinic for Conservative Dentistry and Periodontology, Martin-Luther-University Halle-Wittenberg, University Medicine Halle, Halle, Germany

**Keywords:** oral health status, DMFT, periodontal health, ice hockey, physical performance on-ice and off-ice, posture, endurance, strength

## Abstract

**Introduction:**

Oral health status (OH) seems to be relevant for the overall health status and common physical performance (PP). Aim: The aim was to explore the associations between OH, days of absence (due to illness and injury), parameters of on-ice and off-ice performance diagnostic, and the match performance (MP) over three seasons.

**Methods:**

Twenty male players (age: 27.7 ± 3.6 years; range: 20–34 years; 2 goalies, 7 defenders, and 11 forwards) from a third league professional ice hockey team were investigated regarding several dental parameters (DMFT, PSI, API, and PBI) by a calibrated examiner. The PP diagnostic contains the ice hockey-specific complex test (IHCT), strength-endurance tests (bench press, plank), and endurance tests on bike (PWC 170, 30 min all-out test) as well as the measurement of postural stability and regulation using posturography. Furthermore, the number of sick days and the MP were collected and assessed over three seasons.

**Results:**

We could not find any relevant (*r* > 0.5) correlations between days of absence and the above-mentioned independent variables. A relevant association was found between PSI and penalty times [*r* = 0.46; 95% CI = (−0.04; 0.78)]. The match performance score was associated with the IHCT total time [*r* = −0.55; 95% CI = (−0.82; −0.07)], especially with transition without puck [*r* = −0.62; 95% CI = (−0.85; −0.18)]. Only one player (5%) reported that oral health had ever had a negative impact on his physical performance. This player had 124 days of absence compared with an average of 58 days for all other players.

**Discussion:**

In the framework of this study, dental health and injury prevention did not correlate with each other. Overall, the dental health of the German ice hockey sample examined could be rated above comparable averages. The test and match data once again prove the IHCT to be a suitable assessment tool.

## 1 Introduction

Based on the fast and dynamic nature of ice hockey (e.g., physical contact among players, collisions with sticks, pucks, and boards), this popular sport, especially in northern Europe and North America, carries a high risk of oral and dental trauma (1). Dental injuries can range from minor fractures to complete tooth loss. It is generally known, and often described and discussed in the international literature, that poor oral health can influence, and in worse cases reduce, physical performance and induce a systemic inflammatory response (2–4). In contrast to the clinical relevance in professional sports, this medical aspect is often neglected and underestimated, especially in adult professional athletes (2, 5).

While physical exercise can be beneficial for systemic health, including periodontal tissues, poor oral health can counteract these benefits and negatively affect well-being (6). Especially in sports, oral health is a critical determinant of physical performance, yet it often goes underappreciated in sports medicine (7). Oral health problems, such as periodontitis and cavities or problems regarding wisdom teeth, can lead to systemic inflammation by releasing bacteria and pro-inflammatory molecules into the bloodstream, which can negatively impact athletic performance and recovery by increasing the risk of musculoskeletal injuries and exacerbating chronic inflammatory conditions (8). Examples include famous soccer players as well as athletes from many other disciplines like hockey, winter sports, and (7, 9, 10). This connection is significant because chronic inflammation is a common factor in athletic injuries and poor recovery, and while exercise can improve oral health, poor oral health can reduce physical performance (7). Recent systematic reviews and meta-analyses reveal a global pooled prevalence of dental caries among athletes of approximately 46% (11), with individual studies reporting rates ranging from 20% to 84%, dental erosion from 42% to 59%, gingivitis from 58% to 77%, and periodontal disease between 15% and 41% (12). In a cross-sectional study of 352 elite UK athletes across various sports, 49% had untreated caries, 41% moderate-to-severe erosion, 77% gingival bleeding or calculus, and 22% periodontal pocketing, and roughly 32% reported oral health had negatively impacted their training or performance (13). Similar findings were echoed in Pakistani elite athletes, where 80% experienced at least one oral complaint, and 29% stated it affected their training or competitive output (14). Furthermore, regarding the impact of dental health on self-reported outcomes by athletes gain more and more attention (6). Studies focusing on the influence of dental and oral health on quality of life evaluated in a large cross-sectional study among elite athletes found oral health-related quality of life is diminished in up to one-third of participants (6, 10).

The correlation of diet, physiology, and behavior underlies this oral disease burden. These specific conditions of professional athletes including adjusted nutrition, mental stress and increased activity might also have an impact on the composition and dysbalance of the oral microbiome resulting in higher risk for dental disease like caries and periodontitis (15–17). The regular consumption of sports drinks, carbohydrate gels, and energy bars exposes dentition to acids and sugars, fueling caries and erosion (18). Training-induced xerostomia and heavy mouth breathing reduce salivary protection, further exacerbating these effects (19). Moreover, periodontal disease can trigger systemic inflammation via cytokines like CRP, IL 6, and TNF α, which have associations with fatigue, slowed recovery, and diminished training adaptation (20). Progression of these dental diseases can affect the entire organism, as bacteria can spread from the oral sites via the bloodstream causing a bactermia, increasing the levels of circulating inflammatory markers, which might negatively influence physical performance (21, 22). Furthermore, other functional oral conditions, malocclusion, bruxism and temporomandibular disorders (TMD), may alter mastication efficiency, cause pain and influence nutrient absorption, factors essential for physical resilience especially during the demands of daily elite training (2, 6, 23, 24). Especially athletes, often reporting functional habits like bruxism during exercise and high physical load, might have an increased risk for TMD (25). Recently published results showed that 10% of the investigated 337 athletes reported TMD symptoms (26). Therefore, all these oral health factors might impact physical performance of athletes.

Ice hockey, as a high-impact contact sport with frequent sprint efforts, exposes athletes to unique oral health challenges. In contact sports such as ice hockey and others (27, 28), mouthguards are highly recommended to prevent accident-related dental injuries (29). Their preventive effectiveness is well documented (30). Mouthguard compliance is surprisingly low: one study of ice hockey players found that only 23% wore mouthguards “always”, while 46% reported never wearing them, and 31% had suffered past oral injuries, including lacerations and fractured teeth (31). Another study noted poor awareness and use of mouthguards among Turkish premier league players. Despite binding regulations, discomfort and subjectively perceived performance losses still deter people from using them (31).

Biomechanical data from youth ice hockey indicate that any mouthguard use lowers concussion odds (adjusted OR: 0.36, 95% CI: 0.17–0.73); off-the-shelf models were associated with a 69% risk reduction (adjusted OR: 0.31, 95% CI: 0.14–0.65). Dental custom-fit professional mouthguards delivered from a dental office showed no further reduction in risk (32).

Still, evidence on concussion prevention is mixed, with systematic reviews indicating inconclusive effects (33), although ice hockey data show full facial protection may lower severity and time loss (34).

Nutritionally, strong evidence links sports dietary patterns to dental disease. Frequent intake of acidic, sugary sports products coupled with dry mouth were shown to increase caries and erosion, while delaying oral hygiene until 30 min after acid exposure and using water or fluoride rinses significantly mitigates these effects (12, 18). A study of 70 athletes and non-athletes reported that athletes showed an increased risk for dental erosion. Among athletes, a significant correlation was found between caries prevalence and the cumulative weekly training time (35).

Epidemiologically, mixed-sport data demonstrate that athletes in team or contact sports are about 2.4 times more likely to have caries and 2.0 times more likely to exhibit erosion compared to endurance athletes (13). Moreover, DMFT indices (Decayed, Missing, Filled Teeth) are recognizably increased in individual and contact sport athletes compared to overall results of the average population, according to comparative cohort studies (35). Elite swimmers and cyclists exhibited dentine-level wear in 85% of cases (36).

The impact on physical performance is multifaceted: oral pain may hinder dietary intake, disrupt sleep, reduce concentration, and force training interruptions (13). Systemic inflammation may compromise recovery, while dental injuries can require emergency care during critical periods (13). For ice hockey players, this effect is compounded by high training demands, collision exposure, and physiological stress—all of which amplify oral health risks and highlight the need for comprehensive preventive measures.

To safeguard both oral and athletic health, the following strategies are supported by evidence: regular dental screenings at least biannually; athlete-centered oral hygiene education; tailored nutrition planning to reduce acidic exposure; custom, comfortable oral devices with rigorous cleaning protocols; and improved protective equipment to encourage mouthguard compliance (12, 13, 18).

In summary, ice hockey players face dual oral health threats from dietary exposures and sport-related trauma. The high prevalence of caries, erosion, periodontal disease, and dental injury in this population can impair performance through pain, systemic inflammation, and training interruptions. Integrating targeted preventive strategies—including nutrition, hygiene, mouthguard use, and routine dental care—into athlete management protocols is vital. Such an approach aligns with a holistic sports medicine model that prioritizes both oral health and peak physical performance.

Finally, based on the completely different performance diagnostic and kind of movement (running vs. skating) of ice hockey compared with field hockey, the results are not transferable.

Therefore, the aim of the investigation was to explore and describe potential associations between oral hygiene, dental health, days of absence (due to illness and injury), test performance parameters (on-ice, off-ice), and match performance. First, we hypothesized that oral health conditions have a relevant impact on the number of days of absence. Secondly, we also assumed a relevant influence concerning the test and match performance.

## 2 Materials and methods

### 2.1 General study design

**Figure 1:**
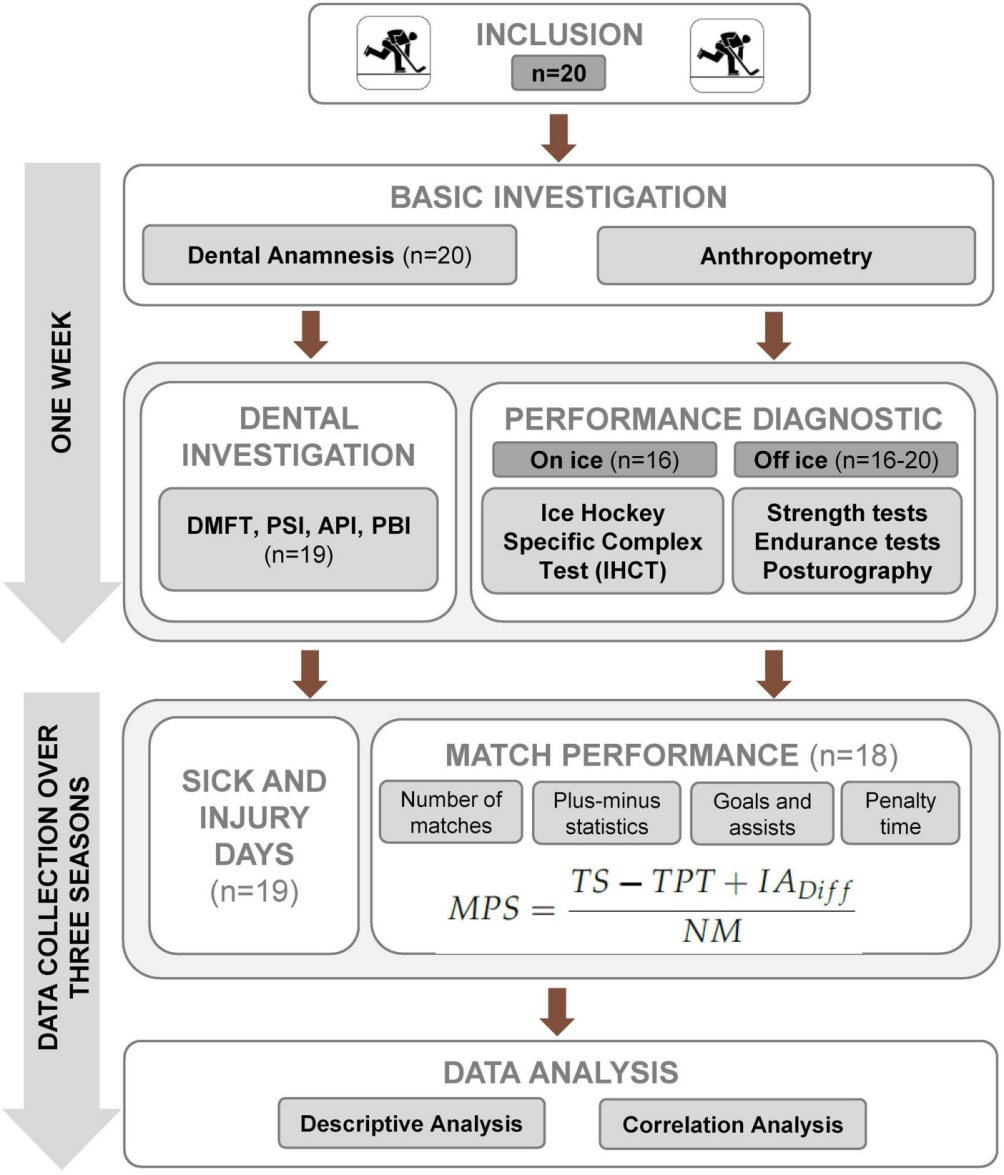
Flow diagram of the study. MPS, match performance score; TS, total score (goals + assist 1 + assist 2); TPT, total penalty time; IA_Diff_, on-ice activity goals for minus on-ice activity goals against.

The data collection was designed as a longitudinal cohort study over three seasons, that is, three years (Figure 1) in order to provide a sufficient sample size.

Over a period of three years, all injuries and illnesses that required sick leave and prevented participation in training sessions and matches were documented and evaluated. Finally, the sum of missing days due to sickness or injury was used for the statistical analyses.

**Figure 2:**
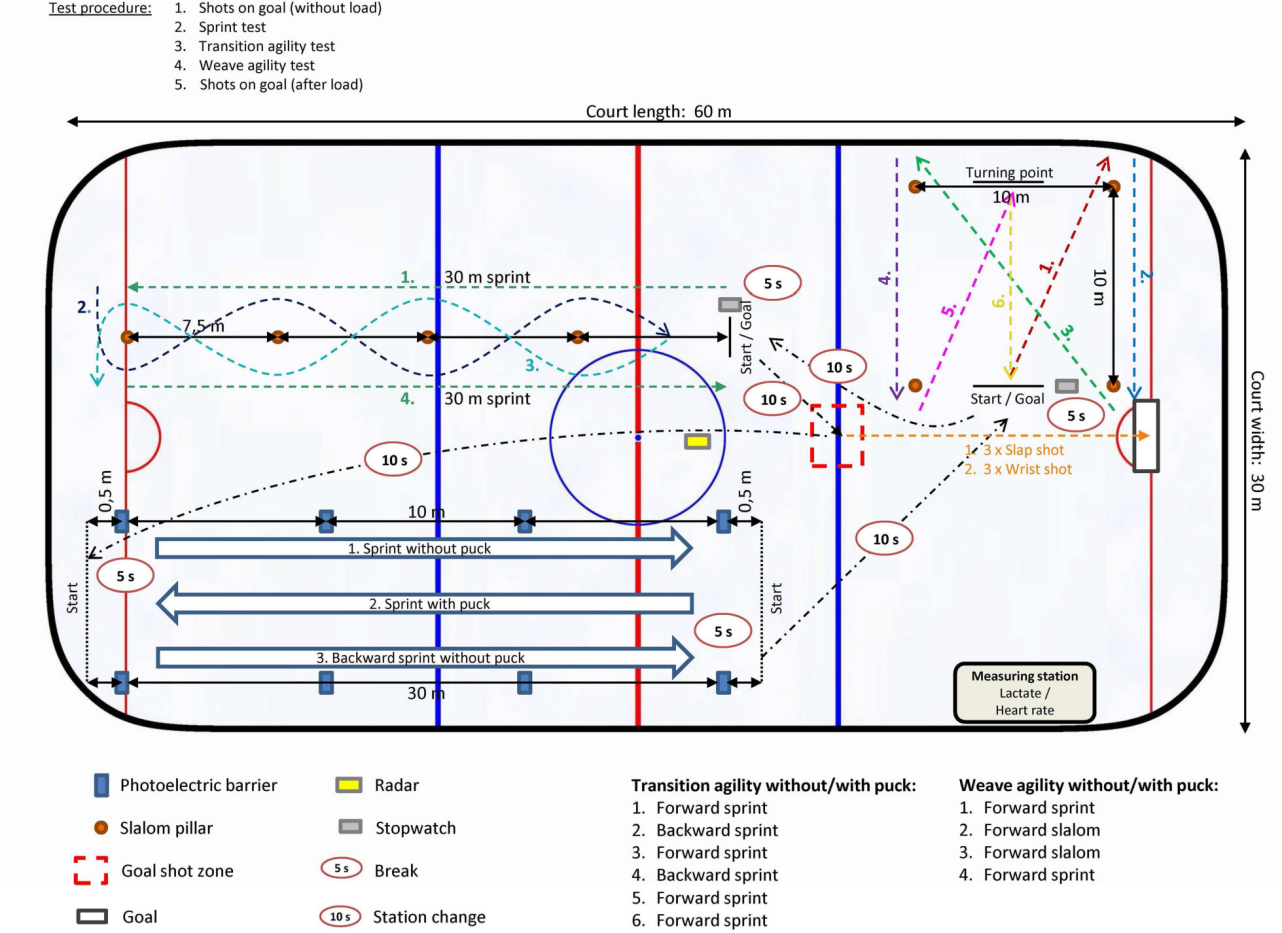
Schematic diagram of the IHCT and description of the test sequences (37).

At the beginning, we conducted a dental anamnesis using a standardized questionnaire and measured anthropometric parameters using a weight scale and stadiometer. In the second step, we conducted a comprehensive performance diagnostic at the beginning of each season. The physical performance diagnostic included several off-ice tests regarding strength endurance (plank, bench press), endurance tests on bike (PWC 170, 30 min all-out test), and postural stability/regulation using posturography (Interactive Balance System; neurodata GmbH, Vienna, Austria) that were conducted in the last week of August in the movement laboratory of the clinic. The IHCT (Figure 2) was connected with the calculation of the MPS (37), the crucial part of the physical performance diagnostic, and was performed in the last week of September. The day before the test day was always a rest day in order to avoid fatigue effects.

The dental examination was carried out by a qualified and experienced dentist. The oral health parameters including the questionnaire were only one time per player at the start of the season.

The study protocol was approved by the local Ethics Committee of the Martin Luther University Halle-Wittenberg (reference number: 2022-011, Date of approval: 18 March 2022) and complied with the Declaration of Helsinki (38). All players (age range: 20–34 years; goalies: *n* = 2, forwards: *n* = 11, and defenders: *n* = 7) were teammates at the beginning of each season of a professional local third league ice hockey team in the eastern part of Germany. All athletes were informed and additionally received written information about the data collection in advance and signed the consent forms indicating their willingness to be part of the investigation. Furthermore, they were motivated to give maximal effort with strong verbal encouragement during all physical performance tests. To ensure high-quality data collection, all athletes were familiarized with the performance diagnostic approach in detail (e.g., verbal instructions, physical demonstration, and presentation via iPad).

### 2.2 Participants

Twenty male elite ice hockey players (Table 1) participated in this study. To ensure high-quality data collection, all athletes were familiarized with the performance diagnostic approach in detail.

**Table 1:** Demographic and anthropometric characteristics of the whole sample (*n* = 20) measured at the start of the preparation phase.

Parameters	Mean ± SD	Median (IDR)
Age [years]	27.7 ± 3.6	27.4 (23.0; 32.8)
Height [m]	1.82 ± 0.07	1.82 (1.70; 1.90)
Body mass [kg]	86.9 ± 9.1	86.9 (75.6; 99.9)
BMI [kg/m^2^]	26.3 ± 1.6	26.2 (23.8; 28.3)
Sick and injured days over three seasons [d]	61.3 ± 42.1	56.0 (11.0; 124)

IDR, interdecile range.

### 2.3 Testing procedures

#### 2.3.1 Questionnaire for oral health conditions

First, the general, medical, and dental anamnesis was taken (Figure 1). A questionnaire regarding oral health conditions using standardized questions was conducted to address specific aspects of professional athletes in ice hockey. The questionnaire to judge the oral health conditions was described in detail in Schwesig et al. (39).

#### 2.3.2 Dental indices and scores

In the present investigation the DMFT Index (D = decayed, M = missing, F = filled, T = teeth) was used to evaluate the cariological and restorative situation regarding the health conditions of the teeth. The used dental indices and scores (DMFT, Decayed, Missing, and Filled Teeth Index; PSI, Periodontal Screening Index; API, Approximal Plaque Index; PBI, Papillary Bleeding Index) are widely used and standardized methods to assess the dental and periodontal situation. They allow an effective examination of the athletes without interfering too much with the training situation in terms of time. Additional x-ray examinations were not performed in this pilot study. Available radiographs from the treating dentists were additionally used if available. The entire approach is already introduced in several scientific works (39–42).

#### 2.3.3 On-ice performance diagnostic—ice hockey-specific complex test (IHCT)

The IHCT was designed to reproduce important match associated actions (e.g., sprints without and with puck, forward and backward, with changes in direction and linear, and slap and wrist shot without and with fatigue) within a complex test design (Figure 2). The goal and substantive justification of the IHCT is to reduce the gap between test and match performance. A detailed description regarding validity (37), reliability (43), and reference data (44) as well as action sequences, used assessments (e.g., lactate and heart rate measurement), and parameters (e.g., definitions and time point of capturing) has already been published before in detail.

To ensure a high degree of standardization, the test preparation (including nutrition, warm-up, and instructions) was comparable for all players, and the same investigators (R.S.: test developer, responsible for correct execution of all actions and time measuring; S.S.: responsible for measurement of heart rate and lactate) performed all test measurements. For the same reason, the ice was reprocessed after half of the players. Furthermore, the investigator (R.S.) gave further instructions during short breaks (5–10 s) between the different test actions when necessary. The rest intervals between the three on ice stations (sprint, transition, weave) was 10 s and within the station (e.g., transition agility without puck and with puck) 5 s.

**Figure 3:**
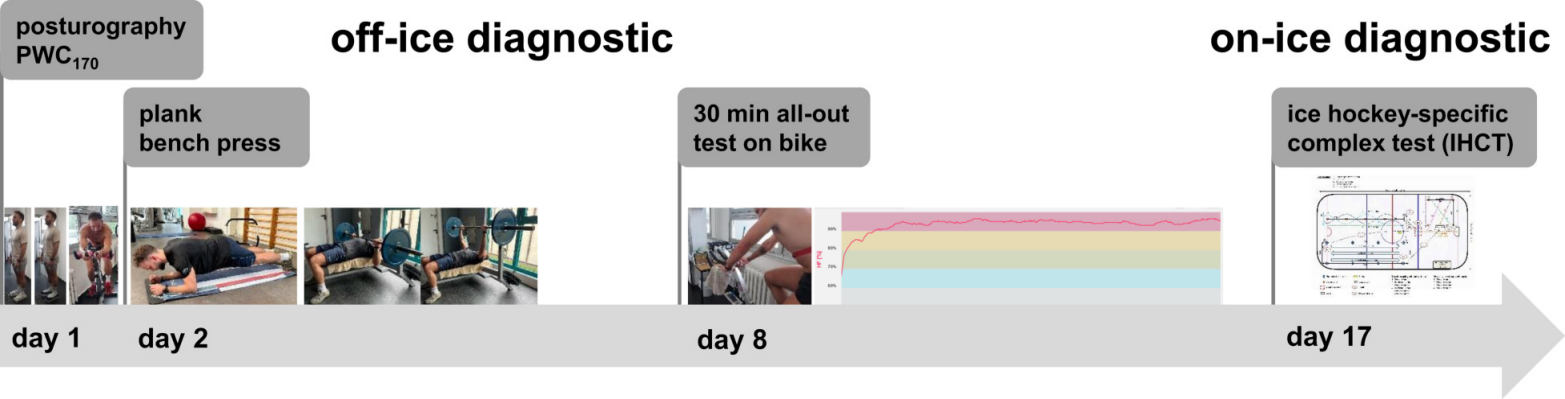
Schedule concerning all off-ice (posturography, plank, bench press, and endurance test on bike) and on-ice (IHCT) diagnostic tests at the beginning of each season.

At the end of the off-ice preseason and preparation phase, the performance diagnostic started with several off-ice tests (Figure 3). The order of tests considered the usually late availability of the rink for on-ice workouts. Therefore, all off-ice diagnostics were conducted before the IHCT. Additionally, adequate resting periods between the several tests were necessary in order to avoid fatigue effects.

#### 2.3.4 Off-ice diagnostic—posturography (posture stability and regulation)

A posturographic measurement system (Interactive Balance System IBS, neurodata GmbH, Vienna, Austria) was used to evaluate postural stability, regulation, and weight distribution. This dynamometric assessment (sampling rate: 32 Hz) was extensively checked regarding validity of the postural subsystems, validity of the postural stability, reliability, and asymptomatic reference data (45). The procedure of the measurement (e.g., instructions and test positions) was also described in detail in the mentioned publications.

#### 2.3.5 Off-ice performance diagnostic—strength tests

The first off-ice test contained front plank to judge the core stability, especially the abdominal endurance. The following execution criteria which were based on several studies (46–48) were implemented:

- No lifting of forearm and feet and 90° flexion in the elbow joint.
- Elbows were vertically below the shoulders, and the forearms remain in a straight position/line (no contact between the hands).
- The neck was kept neutral so that the body remained straight from head to heels.
- Straight in core line (without lifting the buttock).

The test ended when one of the criteria was violated for the second time, or when the player ended the test due to exhaustion (time to exhaustion) or pain that prevented the test position from being maintained.

Secondly, after a suitable resting period (20 min), all players performed bench press in order to evaluate the strength endurance level of the upper body (49). The task was to perform 20 repetitions (upper threshold) with 60 kg in three sets (set rest: 5 min). In the fourth and last set (five minutes later), every player was required to perform the maximal number of individual repetitions. The following execution criteria were controlled:

- Consistent exercise execution without any interruptions;
- Lowering the barbell to the chest (short contact, but no cushioning of the barbell);
- Full extension of the elbow joint;
- Feet placed next to the weight bench;
- No lifting of the core, especially in the thoracic spine area.

#### 2.3.6 Off-ice performance diagnostic—endurance tests on bike

The WHO progressive protocol was used to measure the PWC170; testing on the Emotion fitness cycle 600 med (emotion fitness GmbH & Co. KG/Hochspeyer/Germany). The test started with a loading of 100 W and was increased by 25 W every 2 min. The heart rate was captured continuously by Polar Team Pro System (Polar Electro Sales & Services Europe B.V., Utrecht, Netherlands).

Furthermore, the players' peak endurance performance was tested by a 30 min all-out test on a spinning bike (M3, Keiser Deutschland GmbH, Cologne, Germany). At first, the players performed a standardized 10 min warm up-program, including three 60 s frequency sprints (>100 rpm) after two minutes (resting period: 2 min). An additional criterion for the standardization of the preload was a total energy consumption of at least 100 kcal. The 30 min all-out test started five minutes later. The requirement was to realize the maximum performance output over 30 min using an individual strategy. Heart rate was recorded continuously over the whole test duration. At the end of the test, the lactate concentration of the capillary blood from the earlobe was determined (Super GL Compact; Dr. Müller Gerätebau GmbH, Freital, Germany). Finally, the average wattage was recorded and related to the individual body mass (Watt per kg body mass).

#### 2.3.7 Match performance evaluation

We calculated a match performance score (MPS) for all players as follows (37):

$$MPS=\;\frac{TS-TPT+IA_{Diff}}{NM}$$

**TS** = total score (goals + assists 1 + assists 2);

**TPT** = total penalty time;

**NM** = number of matches;

**IA_Diff_** = on-ice activity goals for minus on-ice activity goals against.

Regarding the judgment of the calculated MPS, the following procedure should be applied: the higher the MPS, the better the match performance. The number of matches over three seasons which was used for the calculation of the MPS ranged from 48 to 171 (mean: 101). A high number of matches reduces the variance and guarantees a high level of MPS validity.

### 2.4 Statistical analysis

All statistical analysis was realized using SPSS version 31.0 for Windows (IBM, Armonk, NY, USA). Descriptive statistics were reported depending on the different scale levels [metric: mean, standard deviation (SD); ordinal: median, percentile (interdecile or interquartile range); IDR, and IQR]. Additionally, skewness (SK) and kurtosis (KU) were reported.

The sample size calculation is based on the estimation of the correlation between parameters of different dimensions (e.g., on-ice performance, dental health, off-ice performance). Assuming a correlation of 0.7 and requiring a 95% confidence of two-sided width of 0.5 [e.g., 95% CI = (0.37; 0.87)] results in a sample size of 36 athletes.

Associations between metric (Pearson's correlation) or ordinal (Spearman correlation) variables were analyzed and interpreted as negligible (<0.1), weak (0.1–0.4), moderate (0.41–0.7), strong (0.71–0.9), and very strong (>0.9) (50). A *r* > 0.7, respectively, *r*^2^ > 0.5 (explained variance > 50%) was defined as relevant. According to the sample size of *n* = 20, the critical value for the product moment correlation based on a two-sided t-test and a = 5% is *r* = 0.423 (51). To control the influence of several factors (age), a partial correlation analysis was performed divide by age as the control variable.

## 3 Results

### 3.1 Characteristics and dental screening results of the included participants

In total, 20 professional ice hockey players were included in the present study. An overview of the investigated sample concerning the main parameters of the several dimensions (e.g., dental health, test and match performance) is presented in Table 2.

**Table 2:** Results of the dental screening and the test and match performance diagnostic.

Dental and match performance parameters	Mean ± SD	Median (IDR)	SK	KU	*n* (%)
Dental health parameters (*n* = 19)					
DMFT	6.37 ± 4.39	5.00 (0.00; 13.0)	0.38	−1.00	
DT	0.37 ± 0.68	0.00 (0.00; 2.00)	1.70	1.71	
MT	0.53 ± 0.91	0.00 (0.00; 2.00)	1.67	1.99	
FT	5.47 ± 3.91	4.00 (0.00; 11.0)	0.36	−1.22	
PSI	2.16 ± 0.61	2.00 (2.00; 3.00)	1.66	4.89	
API (%)	47.3 ± 18.8	47.0 (22.0; 73.0)	−0.18	0.94	
PBI (%)	37.4 ± 19.3	43.0 (11.0; 68.0)	0.14	−1.27	
Traumatic injuries in the mouth					6 (30)
Tooth pain					2 (10)
Tension of the temporomandibular joint					7 (35)
Regular check-ups					8 (40)
Additional oral hygiene procedures					7 (35)
Test (*n* = 16) and match performance parameters (*n* = 18)					
IHCT total time [s]	94.2 ± 3.0	94.2 (90.2; 98.5)	0.16	−1.25	
Plank [s]	315 ± 97.7	304 (180; 453)	0.06	−1.25	
Bench press [*n*]	84.5 ± 11.0	85.0 (67.5; 98.9)	−0.85	1.31	
PWC 130 [W/kg BM]	1.92 ± 0.35	1.86 (1.47; 2.45)	0.36	−0.97	
PWC 150 [W/kg BM]	2.79 ± 0.33	2.78 (2.36; 3.29)	0.09	−1.00	
PWC 170 [W/kg BM]	3.77 ± 0.44	3.88 (3.15; 4.21)	−0.28	−0.65	
30 min all-out test [W/kg BM]	3.22 ± 0.29	3.18 (2.85; 3.66)	0.16	0.30	
Stability indicator (ST)	20.9 ± 6.5	20.2 (14.2; 33.6)	1.25	1.93	
MPS	0.31 ± 0.67	0.32 (−0.88; 1.11)	0.05	1.30	

IDR, interdecile range; SK, skewness; KU, kurtosis.

The dental examination showed an averaged DMFT of 6.37 ± 4.39. However, the restorative status was comparatively high (F = 5.47 ± 3.91). Regarding the specifications of ice hockey as a contact discipline, the number of missing teeth was low (0.53 ± 0.91). The oral hygiene indices of the ice hockey players showed an oral hygiene situation in need of improvement, as the API of the examined players was 47% (±19%) and the PBI 37% (±19%). Only seven of the examined athletes used additional oral hygiene products. The periodontal screening index was 2.16 (±0.61), showing paradontal treatment needs of the included athletes. Six athletes reported traumatic injuries to their teeth, and seven athletes reported problems with the jaw joint and chewing muscles. Regular check-ups and visits to the dental office were reported by eight players. Compared with reference data for the postural stability and regulation, the stability indicator (20.9) moved around the 75th percentile. Consequently, the postural stability of the investigated ice hockey players is slightly below average of an asymptomatic matched (regarding age and gender) sample.

### 3.2 Results of the dental questionnaire and anamnesis

Concerning several oral health statuses, the following aspects were examined using a questionnaire:

• previous illnesses (5%), medication intake (5%), number of sick days in the past two years (not sick: *n* = 2/10%), injured in the past two years (80%), any past operations (70%), any traumatic injuries in the mouth (30%), currently tooth pain (10%), currently bleeding gums (30%), currently grinding teeth (0%), currently tension of the temporomandibular joint (35%), any previously treated orthodontically (35%),regular check-ups (40%), number of teeth brushed per day (two times daily: 85%), additional oral hygiene procedures (35%), satisfaction with oral hygiene (80%), changes in the teeth (40%), and impact on competitive sport (5%).

Eleven (55%) ice hockey players reported five or six diseases in the past two years. Eighty-five percent (*n* = 17) brush their teeth twice a day, and 35% (*n* = 7) practice additional oral hygiene. Only one player (5%) was of the opinion that oral health had a negative impact on his physical performance. This player had 124 days of absence (sickness and injuries) compared with an average of 58 days for all other players. Table 3 contains a descriptive analysis of the dental anamnesis in relation to its importance for days of absence.

**Table 3:** Sick and injured days in relation to selected aspects of the dental anamnesis (*n* = 19).

Aspects of the dental anamnesis	Sick and injured days; Mean ± SD	
	Yes	No
Do you have toothache?	*n* = 2: 77 ± 79	*n* = 17: 60 ± 40
Did you have gum bleeding in the past?	*n* = 6: 83 ± 52	*n* = 13: 51 ± 35
Do you suffer from tensions around your jaw joint/shoulder/neck?	*n* = 7: 72 ± 47	*n* = 12: 55 ± 40
Did you undergo any orthodontic treatments?	*n* = 7: 59 ± 52	*n* = 12: 63 ± 38
Do you regularly go to the dentist?	*n* = 8: 52 ± 38	*n* = 11: 68 ± 38
Do you use any additional oral care products?	*n* = 7: 54 ± 43	*n* = 12: 66 ± 43
Are you happy with your oral health?	*n* = 15: 57 ± 40	*n* = 4: 79 ± 50

### 3.3 Associations between sick and injured days over three years, oral health, test and match performance parameters

The number of days of absence due to illness and injury, especially over a long period, is a valuable and crucial indicator of the health status of every player. Therefore, we calculated possible associations with different oral health, test and match performance parameters taking into account the influencing variable of age. We could not find any relevant correlation of practical value (*r* > 0.7). Concerning the dental indices and scores, the largest association was calculated with the MT [*r* = 0.46; 95% CI = (−0.01; 0.76)]. Concerning the test performance diagnostic, the largest amount was found for weave agility without puck [*r* = −0.43; 95% CI = (−0.76; 0.09)] and PWC 130 [*r* = 0.41; 95% CI = (−0.06; 0.73)]. Concerning match performance and dental health, the largest association was observed for PSI and penalty times [*r* = 0.46; 95% CI = (−0.04; 0.78)] without any causality being able to be inferred from this. This was the only association that was virtually unaffected by age, whereas the other three correlations showed a clear age effect (Table 4).

**Table 4:** Partial correlation (control variable: age) in order to clarify associations between parameters of different dimensions sorted in descending order based on the total correlation.

Parameters	*r*	95% CI	Partial correlation
PSI vs. penalty times	0.46	−0.04–0.78	0.51
Number of days of absence due to illness and injury vs. MT	0.46	−0.01–0.76	0.17
Number of days of absence due to illness and injury vs. weave agility without puck	−0.43	−0.76–0.09	−0.15
Number of days of absence due to illness and injury vs. PWC 130	0.41	−0.06–0.73	0.26

### 3.4 Associations between test and match performance parameters

Of secondary importance was the judgment of associations between test and match performance parameters. The match performance score (MPS) as indicator for the global match performances showed the following noteworthy but not relevant correlations concerning several physical performance parameters:

- Transition agility without puck: *r* = −0.62; 95% CI = (−0.85; −0.18);
- IHCT total time: *r* = −0.55; 95% CI = (−0.82; −0.07);
- Weave agility without puck: *r* = −0.51; 95% CI = (−0.80; −0.02).

**Figure 4:**
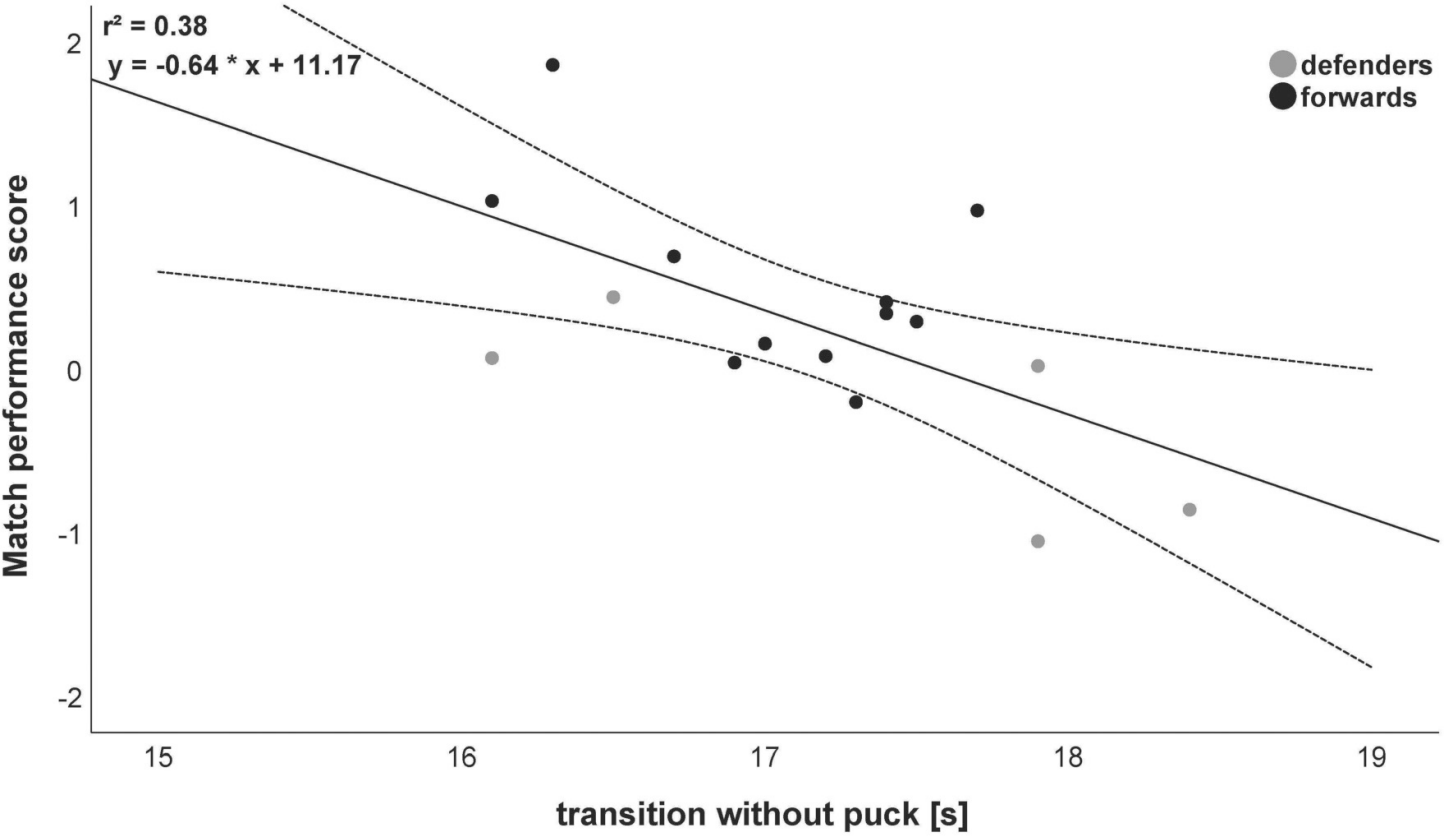
Association between match performance score (MPS) and transition without puck. Please note that one dot can represent several subjects. The solid line represents the linear relationship between MPS and transition without puck. The dashed lines indicate the 95% CI.

Consequently, the transition without puck performance is able to explain 38% of the variance of the MPS (Figure 4).

Regarding single match performance parameters, the number of points per matches displayed the largest number of relevant correlations to the following IHCT parameters:

- Weave agility without puck: *r* = −0.64; 95% CI = (−0.86; −0.22);
- IHCT total: *r* = −0.59; 95% CI = (−0.84; −0.13);
- 30 m sprint with puck: *r* = −0.55; 95% CI = (−0.82; −0.08).

The transition agility without puck showed the largest number of relevant correlations of the single IHCT parameters concerning posturography parameters:

- F5–6 (somatosensory system): *r* = −0.63; 95% CI = (−0.86; −0.20);
- F1 (visual and nigrostriatal system): *r* = −0.55; 95% CI = (−0.82; −0.08);
- F7–8 (cerebellar system): *r* = −0.55; 95% CI = (−0.82; −0.07);
- ST (stability indicator): *r* = −0.53; 95% CI = (−0.81; −0.05).

## 4 Discussion

### 4.1 Summary of the main findings

This is one of the first data collections to investigate the global health status measured by the days of absence of elite ice hockey players based on different influence factors, especially the oral health conditions. Additionally, the on-ice and off-ice performance were related to the match performance, and both dimensions were associated with global and oral health. There is currently no comparable complex data collection with adult ice hockey players in the elite class, which makes it difficult to discuss the results.

### 4.2 Hypothesis-based discussion: oral health status and physical performance

Based on the high level of oral health (DMFT: 6.4; PSI: 2.2; API: 47%; and PBI: 37%) connected with the low number of abnormalities and complaints (e.g., tooth pain: 10%; traumatic injuries in the mouth: 30%; and tension of the temporomandibular joint: 35%), we could not find any relevant correlations with the days of absence.

The oral health results displayed that the examined elite ice hockey players had some deficits, for example, compared to soccer players (52). In particular, the DMFT values (6.4 ± 4.4) indicate a considerable high caries burden. In line with the oral hygiene parameters (API, PBI) and the periodontal values (PSI), the present results show that the included German ice hockey players are well rehabilitated, and there is little need for additional dental treatment. The F-value (5.47 ± 3.91), representing the restored teeth, was also very high and accounts for more than 90% of the total DMFT value. The M-value (0.53 ± 0.91), representing missing teeth, especially when considering the fact that ice hockey is a high-risk discipline for dental trauma, was surprisingly low. The results are comparable with the findings from other authors (12, 42) concerning caries prevalence and periodontal status. Schulze/Busse et al. (12) reported untreated dental caries in 29% of adults and a DMFT of 6.7 (ages 20–34 years) based on a comprehensive data collection from The National Institute of Dental and Craniofacial Research Health. DMFT values of elite soccer and rugby players (ages 21–27 years) ranged from 2.7 to 5.7 (9, 52, 53) as a sign of a higher level of oral health. Gallagher et al. (23) investigated 352 athletes in a comparable age (mean age: 25 years; range: 18–39 years) from eleven sports (e.g., hockey, *n* = 46). The most commonly reported impacts were oral pain (30%) and difficulties with eating (35%) (23). In our study, the percentage of players with oral pain was significantly lower (10%). On the other hand, we observed the same percentage of players, which felt that their performance was affected by oral health (5% vs. 6%). Gallagher et al. (23) described that 69% of athletes rated their oral hygiene as good/very good compared with 80% of ice hockey players in the present study. Sevindik et al. (54) also detected a similar oral health for ice hockey players. According to DMFT, the markedly younger ice hockey players (*n* = 22, age: 15.1 ± 0.4 years) displayed similar results (5.90) compared with our investigated sample (median: 5.00; mean: 6.4).

A crucial aspect of this work was to explore possible associations between oral health and physical performance parameters with the number of sick and injured days. Gay-Escoda et al. (52) evaluated the oral health status of professional Spanish soccer players (*n* = 30) and its relation to the incidence of sport lesions. The authors found significant but not relevant correlations between Hein plaque index (PI; *r* = 0.42) and Ramfjord teeth probing pocket depth (PPD, *r* = 0.39) to intrinsic muscle injuries. We also could not find any relevant (*r* > 0.7) correlations between oral health scores and parameters and the number of sick/injured days. The largest correlation coefficient was calculated for a single on-ice parameter of the IHCT (weave agility without puck: *r* = −0.43; 95% CI = (−0.76; 0.09). Consequently, we must reject the central hypothesis (oral health conditions have a relevant impact on the number of days of absence) of this work. Furthermore, in the present study periodontal and caries indices in form of the DMFT and PSI index were used in this pilot study. These indexes are used in many investigations focusing on oral health conditions. However, the use of ICDAS or EA4SD criteria limits comparability with contemporary sports dentistry literature. Therefore, this might be a limitation of the present study and should be taken into account when examining a larger number of ice hockey players in order to possibly re-examine the hypothesis rejected in this study.

Despite the rejected hypothesis in the present study, numerous reviews identified oral (cariological and periodontal) pathologies as prevalent in elite athletes and connected to systemic inflammation potentially impairing exercise capacity and performance (2, 6, 12, 55). The linking mechanisms between poor oral health and performance are multifaceted. Chronic dental infections elevate systemic cytokines (e.g., IL-6, TNF α), which contribute to muscle fatigue, oxidative stress, and impaired recovery (56, 57). These conditions of professional athletes including adjusted nutrition, mental stress and increased activity might also have an impact on the composition and dysbiosis of the oral microbiome resulting in higher risk for dental diseases like caries and periodontitis (15–17). Progression of these dental diseases can affect the entire organism, as bacteria can spread from the oral sites throughout the organism via the bloodstream causing a bacteremia, increasing the levels of circulating inflammatory markers might negatively influence physical performance (21, 22). Further evidence points to sport-specific behavior that exacerbates oral disease. Repeated consumption of acidic carbohydrate-rich sports and energy drinks was linked to enamel erosion and caries in athletes (14). Reduced salivary flow during intense exercise common among endurance-focused players, combined with inadequate sugar intake further elevates susceptibility to tooth decay (58, 59). Despite adequate oral hygiene procedures like teeth brushing being common among athletes, many fail to counterbalance dietary and training-related risks (58).

Biological markers support these findings. Studies show links between periodontal inflammation and elevated C-reactive protein and TNF α levels, factors associated with impaired muscle regeneration and slower recovery (60). Furthermore, dental screening in elite athletes has uncovered associations between gingivitis severity and time lost to injury (61). In light of these findings, preventive strategies are being explored and demonstrated that implementation of tailored oral health education among professional athletes improved periodontal outcomes and reduced inflammatory biomarkers (62). Meanwhile, integrating dental care into high-performance programs underlines the growing recognition of the oral health and physical performance connection (63). Compared to general studies including not only elite athletes or soccer players the overall global oral health situation is still challenging (64). In Germany, actual studies show positive developments regarding oral health conditions in general (65). However, in spite of proportional reductions, the polarized caries distribution following socio-economic parameters reveals the need for specialized preventive care concepts also in professional and international composed teams in sports (55, 66, 67).

### 4.3 Hypothesis-based discussion: off-ice and on-ice physical performance

Based on the reference data (*n* = 138) from Schulze et al. (44) concerning the IHCT, the on-ice performance level of the investigated ice hockey players can be rated as good to very good. The IHCT total time (94.2 s) moved slightly above the 25th percentile (93.8 s) and markedly below the median (95.5 s) and mean (96.1 s).

The MPS (0.31) as indicator for the match performance was clearly lower than in the study of Schwesig et al. (37). This validation data collection provided a mean value from 0.42 (*n* = 17).

The measured postural stability was markedly lower than by Schwesig et al. (68) because the stability indicator was markedly higher (20.9 ± 6.52 vs. 17.2 ± 3.43; *d* = 0.74). In relation to asymptomatic reference data [age range: 20–30 years, men; (45)], the stability indicator moved around the 75th percentile (20.4). That means that the investigated ice hockey players belong to the weakest 25% of their age group and gender. Especially, the high level of weight distribution in both directions (anterior–posterior and medio–lateral) and foot coordination (parameter: synchronization) compared with soccer players and normal subjects is remarkable. Presumably responsible for this are ice hockey specific maneuvers like turns or checks. The players move on very narrow skate blades (width: 3 mm; length: 254–282 mm) and have to control their strength very precisely in order to change the direction or accelerate/decelerate without and with body contact.

The endurance performance measured on bike (PWC 130, 150, 170) was on all levels higher than by Schwesig et al. (68). The largest difference (*d* = 0.76) was calculated for PWC 170 (3.77 ± 0.44 W/ kg BM vs. 3.38 ± 0.59 W/kg BM).

Cordingley et al. (48) tested 103 competitive youth ice hockey players for three consecutive years (13, 14, and 15 years of age) regarding different dimensions (strength, endurance, agility, and core stability). In line with our investigation, plank was used to judge the core stability of the ice hockey players. The observed performance level was much lower (107–115 s) than in our adult sample (315 ± 98 s). Surprisingly, maximal plank time significantly decreased (d = 0.47) between the ages of 14 (115 ± 13 s) and 15 (107 ± 21 s) years (48). Tong et al. (47) investigated university athletes (*n* = 36; age: 22.4 ± 3.7 years) using an endurance plank test. The average time to exhaustion was also strongly lower (174 ± 49 s) than in our examined sample. Schellenberg et al. (46) evaluated the static trunk flexor muscle endurance in healthy non-athletic male adults (*n* = 22; mean age: 34 years). The captured time of exhaustion (93 ± 29 s) was comparatively low. All reported plank results underline the high physical performance level of our investigated ice hockey players, especially according to the core stability.

Nordström et al. (69) examined 133 male ice hockey players (mean age: 24 years, range: 17–41 years) from the GET league (premier professional league in Norway) concerning different physical performance dimensions. Regarding bench press as indicator for the maximum upper body strength (pectoralis major, triceps, and anterior deltoid muscles), a one repetition maximum (1-RM) of 107 kg (range: 80–145 kg) was captured. According to the recalculation of our strength endurance set up based on the Holten curve (70) (mean number of reps: 20 ≈ 72%; minimum of reps: 15 ≈ 76%; maximum of reps: 40 ≈ 47%), a mean value from 80 kg (range: 75–133 kg) was estimated. It should be noted that the accuracy of the estimate (percentage of intensity) decreases as the number of repetitions increases. Unfortunately, Nordström et al. (69) did not report any information to the body weight of the athletes. Therefore, it is not possible to calculate the relative strength of the upper body, which limits the comparability of the results. In line with our results, Nordström et al. (69) stated that a low physical performance level is not associated with an increased rate of injury and illness.

### 4.4 Limitations

The data collection has some relevant limitations. Firstly, the most important limitation is the lack of such complex (oral health, match performance, off-ice and on-ice test performance, analysis of sick and injury days) scientific investigations with adult professional ice hockey players in the field of performance diagnostic. Of course, also a sample of 20 hockey players may negatively affect the reliability of the data obtained and the substantially limits generalization. Based on the literature and our own experiences, it is very difficult to convince and win the responsible persons (manager, coach, and team doctor) regarding the necessity of such complex and longitudinal data collection. Therefore, effective and sport adjusted protocols might be helpful. There are recently published assessment tools available to screen oral health conditions in sports (71). The introduced Universal Screening Protocol for Dental Examinations in Sports (USPDES) might be a suitable alternative for further studies in larger populations.

Secondly, regarding the IHCT, this test was originally only developed for field players. Therefore, the data of the goalies (*n* = 2) are missing in this context. Thirdly, future studies should focus more on the age aspect. Under certain circumstances, age-related changes can be determined by practicing the sport. For this reason, it is necessary to increase the sample size or compare a junior team with a professional team. Moreover, further control and confounding variables (e.g., sleep quality, nutrition, diet logs, training loads, etc.) could not be considered and therefore should be included in future study designs.

The aerobic performance is a crucial performance factor in team sports (e.g., ice hockey, soccer). Surprisingly, we found a direct correlation between PWC 130 and the number of days of absence due to illness and injury. Presumably, a more detailed analysis of playing time and the kind of injury (e.g., contact or non-contact; during the match or training) in future studies is necessary for a final judgement.

Finally, also the qualification of the ice hockey players (third vs. first league) could be a distinguishing feature for all or some of the variables examined. Another limitation might be that only one team was investigated. Therefore, general conclusions should be made with caution. Further studies, comparing different teams, regions, and leagues should be carried out to allow recommendations for ice hockey team athletes.

## 5 Conclusions

In conclusion, this pilot study provides a valuable, comprehensive overview regarding global health, dental health status, oral hygiene performance, physical performance (off and on-ice), and match performance for German elite ice hockey players over three seasons. Based on these data, every coach and player has the possibility to compare their own data with the presented dental health and physical performance data.

The primary outcome “days lost due to illness and injury” seems not to be associated with the above-mentioned parameters from different dimensions. An increase in the sample size is necessary for a conclusive statement and to extend the scope of validity. Subsequently and based on the results of the partial correlation analysis, an age-stratified analysis may also be possible.

Specific screening protocols, like biannual dental checks (e.g., tooth vitality, traumatic injuries, cariological, periodontal, and restorative screening) tailored for ice hockey teams, should be part of a comprehensive medical (e.g., orthopedic and cardiological) and physical (e.g., posture, strength, endurance, and IHCT) health and performance check. A possible risk for sports-dependent caries prevalence requires risk-adapted preventive oral health concepts in the field of sports dentistry.

In the future, multicenter, multisport longitudinal studies will be necessary to investigate and clarify possible multifactorial relationships based on larger sample sizes.

## Data Availability

The raw data supporting the conclusions of this article will be made available by the authors, without undue reservation.
